# Association between self-reported periodontitis and high-risk oral human papillomavirus infection among Indigenous South Australians: A cross-sectional study

**DOI:** 10.1371/journal.pone.0265840

**Published:** 2022-03-24

**Authors:** Anna Ali, Alice R. Rumbold, Kostas Kapellas, Zohra S. Lassi, Joanne Hedges, Lisa Jamieson

**Affiliations:** 1 Robinson Research Institute, School of Medicine, The University of Adelaide, Adelaide, South Australia, Australia; 2 Australian Research Centre for Population Oral Health, The University of Adelaide, Adelaide, South Australia, Australia; 3 South Australian Health and Medical Research Institute, Adelaide, South Australia, Australia; All India Institute of Medical Sciences, INDIA

## Abstract

**Introduction:**

The incidence of oropharyngeal squamous cell carcinoma (OPSCC) is increasing globally, reflecting an increase in human papillomavirus (HPV)-related lesions. Indigenous populations are disproportionately affected by OPSCCs. Currently, testing for oral HPV is not recommended as a screening tool to permit early detection of OPSCCs due to the high population prevalence of HPV infection. Periodontitis may be a marker of oral HPV infection, but previous research evaluating this association has been inconclusive. Here we report a large population-based study examining the association between high-risk oral HPV infection and periodontitis among Indigenous South Australians.

**Methods:**

We utilised a large convenience sample of Indigenous South Australians aged 18+ years recruited between February 2018 and February 2020. Of the original cohort (n = 1011), 748 (73.9%) participants participated in the 12 month follow-up. Detailed information on sociodemographic characteristics, health-related behaviours, and sexual history were collected at enrolment. Saliva samples were collected at 12 months and tested for the presence of oral HPV DNA using the optimized general primer (GP) + PCR system. The primary outcomes were the prevalence of any high-risk oral HPV DNA, and separately, HPV 16 and/or 18. Periodontitis was assessed at follow-up by using validated self-reported periodontitis screening questions. Logistic regression analyses were undertaken to assess the association between self-reported periodontitis and oral HPV infection with adjustment for potential sociodemographic and behavioural confounders, with estimates presented as odds ratios (OR) and 95% confidence interval (CI).

**Results:**

Data on 673 participants (89.9% of the follow-up cohort) were available. Participants ranged in age from 18 to 80 (mean age 42.2, SD 14.7) and 31.5% were male. Overall, 115 (17.1%) participants had self-reported periodontitis, 40 (5.9%) had any high-risk oral HPV and 14 (2.1%) had HPV 16 and/or 18. Any high-risk HPV was detected among seven (17.5%) participants and HPV 16 and/or 18 was detected in three (21.4%) who self-reported periodontitis. In the regression analyses no significant association was found between self-reported periodontitis and high-risk oral HPV (adjusted OR: 1.10; 95% CI: 0.45–2.70) or HPV 16 and/or 18 (adjusted OR: 1.27; 95% CI: 0.32–5.03).

**Conclusion:**

This study did not find any association between self-reported periodontitis and high-risk oral HPV among Indigenous South Australians. Further targeted studies with standardized clinical measures of periodontal disease are needed to clarify the link between high-risk oral HPV and periodontal disease. If confirmed this would add further weight to the importance of recommendations about the utility of periodontitis screening to identify individuals at risk of carrying high-risk oral HPV, who may benefit from more intensive screening and ongoing monitoring.

## Introduction

The global prevalence of human papillomavirus (HPV)-associated oropharyngeal squamous cell carcinoma (OPSCC) is between 23.0–31.0% [1, 2], and there is evidence in many settings that the incidence of HPV-positive OPSCC is increasing [3]. While known risk factors for OPSCCs include smoking and alcohol consumption [4], in the past decade there has been a dramatic change in the epidemiology of OPSCCs, with a reduction in OPSCCs caused by tobacco (reflecting reduced smoking rates), and a steady increase of this cancer in younger age groups, primarily due to oncogenic HPV infection [3, 5, 6].

Currently, screening for OPSCCs is done visually via an examination of the oral cavity to detect cancerous lesions. While screening has been shown to improve clinical outcomes only in those who are at risk of oral cancer [7, 8], many professional dental organisations recommend routine screening as part of a comprehensive oral examination [9]. However, there remains considerable debate about the value of screening individuals without established risk factors, namely, tobacco and heavy alcohol use [10]. In the case of HPV-positive OPSCC, it is difficult to identity a specific population at risk, and testing for oral HPV DNA is not recommended due to the widespread exposure to HPV in the population, and current inability to reliably determine which individuals who are oral HPV-positive will go on to develop malignant lesions. This lack of understanding about the natural history of oral HPV infection and the carcinogenic process is a major impediment to improving early detection of HPV-positive OPSCC.

Nearly two decades ago, the presence of high-risk HPV in healthy and diseased periodontium was reported [11]. Recent systematic reviews have reported a consistent association between periodontitis with any oral HPV infection, but the association with high-risk HPV is inconclusive [12, 13]. Further, targeted research among populations at risk of OPSCCs is needed to understand the relationships between HPV, periodontitis and OPSCCs.

In Australia, 3.3% of the population are Indigenous, which includes Aboriginal and Torres Strait Islander peoples with unique languages, histories, and cultural traditions. Like many other health conditions, Indigenous Australians experience a disproportionate burden of poor oral health. Both incidence and mortality from OPSCCs are considerably higher in this group than in the Australian non-Indigenous population [14]. Similarly, rates of periodontal disease are significantly higher among Indigenous communities compared to the non-Indigenous population, and the Indigenous population is more than twice as likely to have advanced periodontal disease [15]. Recent data also confirms a high prevalence of oral HPV in this population [16].

There has been limited examination of the possible link between high-risk oral HPV and periodontitis among Indigenous Australians, despite the high burden of oral health conditions and OPSCCs in this population. If a link between oral HPV infection and periodontitis is confirmed, then this population may have great potential to benefit from improved screening for periodontitis and high-risk HPV infection. The aim of this study was to assess the association between periodontitis and high-risk oral HPV among Indigenous South Australians. We hypothesized that individuals with periodontitis would have higher prevalence of high-risk oral HPV compared to those with no periodontitis.

## Methods

### Study design and participants

We used a large convenience sample of Indigenous South Australians aged 18+ years recruited between February 2018 and February 2020 as part of a broader study investigating oral HPV and OPSCCs among Indigenous Australians [16, 17]. Indigenous people living throughout South Australia were recruited and followed-up at 12 months. At baseline, data were obtained for 1011 participants and 748 were followed-up at 12 months. The current study utilises information collected at the baseline assessment and 12 month follow-up. The study was supervised by an Indigenous Reference Group and trained Indigenous research officers collected the data from participants who were recruited through local Aboriginal Community Controlled Health Organisations (ACCHOs).

An Indigenous research officer explained the study aims and objectives to the participants and after obtaining written consent, participants were asked to share information related to their sociodemographic characteristics, health-related behaviours including tobacco and alcohol use, sexual history, self-reported oral and general health, HPV vaccination and answer questions about self-reported periodontitis. Each participant was requested to provide a saliva sample, through spitting and dribbling in a commercially available kit (Omnigene OM-501; DNA Genotek Inc, Canada) from which microbial DNA for genotyping was extracted.

### Self-reported data

Sociodemographic information collected at baseline included age, gender, geographic location, highest level of education completed and sexual history. Information on health-related behaviours including tobacco consumption, alcohol intake, and self-reported periodontitis were derived from the 12-month follow-up.

Age was dichotomised into individuals aged up to 40 years and 41 or older. Geographical location was categorised as metropolitan or non-metropolitan. The highest completed education level was dichotomised as up to and including high school or tertiary level. Smokers were categorised as a current regular smoker or non-smoker. Alcohol consumption included ex/current alcohol use or never consumes alcohol. Oral sex history was categorised as yes or no.

### Laboratory analysis

Saliva samples were collected using a commercially available kit (Omnigene OM-501; DNA Genotek Inc., Canada), and analysed for HPV status using the optimized general primer (GP)+PCR system, which detects most mucosal HPV types and all high-risk HPV types. All HPV DNA positive samples were sequenced to confirm viral DNA sequences. Detail on the laboratory processes has been published elsewhere [16]. For the current study, HPV status was taken from lab analysis at 12 months follow-up.

### Exposure: Self-reported periodontitis

At 12 month follow-up, self-reported periodontitis was assessed using eight periodontitis screening questions which have previously been validated among non-Indigenous Australians as part of the Australian National Survey of Adult Oral Health (2004–2006) administered via questionnaire [18]. The eight questions ascertain self-reports of gum disease, bone loss, history of scaling/root planning, loose teeth, use of mouth wash and dental floss and overall gum health. In the previous validation studies, four questions were significantly associated with clinically assessed moderate and severe periodontitis, these included: 1) Do you think you have gum disease? 2) Have you ever been told that you have lost bone around your teeth? 3) Have you ever received scaling and root planing? and 4) Have you ever had any teeth become loose on their own, without an injury? Individuals with affirmative responses to all of these questions were classified as having periodontitis. Those responding ‘no’ to any of the above questions were categorised as not having periodontitis.

### Key outcome: High-risk HPV and HPV 16 and/or 18

There were two main outcomes: first, the presence of any high-risk oral HPV (Yes/No); and second, the presence of two specific subtypes, HPV 16 and/or 18 (Yes/No). Individuals were classified as having any high-risk HPV if they had a saliva sample positive for any one of the following HPV subtypes: 16, 18, 31, 33, 34, 35, 39, 45, 51, 52, 56, 58, 59, 66, 68, and/or 70 in line with IARC classifications [19]. For oral cancers HPV 16 and HPV 18 are of primary concern and therefore these were studied separately.

### Statistical analysis

Descriptive analyses were undertaken to ascertain frequencies of all eight questions on self-reported periodontitis as well as the frequency of any high-risk oral HPV, and separately, any HPV 16 and/or 18 subtypes. Bivariate analyses were initially conducted to explore relationships between oral HPV status and sociodemographic status, health-related behaviours, sexual history characteristics and periodontitis. Logistic regression models were undertaken to examine the association between self-reported periodontitis and separately, high-risk oral HPV and HPV 16 and/or 18. Estimates are reported as an odds ratio (OR) with 95% confidence interval (CI). Models were adjusted for potential confounders reported in the literature [20–22] along with all the variables with p ≤ 0.25 in the bivariate analyses. Two separate multivariate models were developed: Model A represents the association between periodontitis and any high-risk oral HPV (IARC classification) and Model B represents the association between self-reported periodontitis and HPV16 and/or 18. Both Model A and Model B included adjustment for age, gender, location, education, smoking, alcohol intake and history of oral sex. Model performance was evaluated by estimating model discrimination (c-statistic) and calibration. All analyses were conducted using STATA 15.

### Ethics

This study was granted ethics approval from the University of Adelaide Human Research Ethics Committee and the Aboriginal Health Council of South Australia’s Human Research Ethics Committee (H-2016-246). Interviewers (Indigenous and non-Indigenous) with extensive experience of working with Indigenous Australians conducted the surveys at each time point. Participant information sheets were shared with the participants in plain English language before commencing the survey. Participants provided written informed consent.

## Results

Data on oral HPV status and periodontitis status were available for 673 participants (89.9% of the follow-up cohort) at the follow-up. Regarding sociodemographic characteristics, just over half were aged 40 years or younger (50.5%), more than two thirds were females (68.5%), living in non-metropolitan location (65.9%), and had attained a high school-level education (66.6%). Three quarters of participants were smokers (74.7%) and 91.2% had currently or previously consumed alcohol. Sixty five percent of participants reported a history of oral sex (Table 1). Overall, 17.1% had self-reported periodontitis, 5.9% had any high-risk oral HPV and 2.1% had HPV 16 and/or 18. There was no difference in the prevalence of periodontitis with respect to age, gender, education, smoking status, alcohol intake, history of oral sex and HPV status (Table 1).

**Table 1:** Description of the study participants (N = 673).

Study variables	Total	Periodontitis	
	N = 673 (%)	Yes	No
		n = 114(%)	n = 547(%)
Age			
≤40	340 (50.5)	66 (19.4)	274 (80.6)
>40	333 (49.5)	49 (14.7)	284 (85.3)
Gender			
Male	212 (31.5)	34 (15.9)	179 (84.1)
Female	461 (68.5)	81 (17.7)	379 (82.3)
Location			
Non-metropolitan	444 (65.9)	74 (16.7)	370 (83.3)
Metropolitan	229 (34.1)	41 (17.9)	188 (82.1)
Completed education level¥			
High school or less	440 (66.6)	69 (15.7)	371 (84.3)
University or further	221 (33.4)	45 (20.4)	176 (79.6)
Tobacco smoking¥			
Yes	495 (74.7)	88 (17.8)	407 (82.2)
No	168 (25.3)	26 (15.5)	142 (84.5)
Alcohol intake			
Ex-drinker/Current	614 (91.2)	105 (17.1)	509 (82.9)
Never	59 (8.8)	10 (16.9)	49 (83.1)
History of oral sex¥			
Yes	388 (64.7)	75 (19.3)	313 (80.7)
No	211 (35.3)	28 (13.3)	183 (86.7)
Any high-risk HPV			
Yes	40 (5.9)	7 (17.5)	33 (82.5)
No	633 (94.1)	108 (17.1)	525 (82.9)
HPV 16 and/or 18			
Yes	14 (2.1)	3 (21.4)	11 (78.6)
No	659 (97.9)	112 (17.0)	547 (83.0)

¥ Missing values

In the bivariate analyses, being ≤40 years and living in metropolitan location were both significantly associated with the presence of any high-risk oral HPV (OR: 2.12; 95% CI: 1.08–4.19 and OR: 2.80; 95% CI: 1.47–5.36, respectively). Regarding HPV 16 and/or 18, there was a significant association between age and the presence of HPV 16 and/or 18 (OR: 3.67; 95% CI: 1.02–13.30).

In the unadjusted models, there was no significant increase in odds of either any high-risk oral HPV or HPV 16 and/or 18 among individuals with self-reported periodontitis (OR 1.03, 95% CI: 0.44–2.39 and OR 1.33, 95% CI: 0.37–4.85, respectively) (Tables 2 and 3). The findings were unchanged in the models that included adjustment for age, gender, location, education, smoking status, alcohol intake and history of oral sex (Table 4). Both adjusted models had moderate discrimination (C-statistic of 0.72 and 0.81 respectively) and good model calibration (S1 Fig).

**Table 2:** Bivariate association between sociodemographic and periodontitis with any high-risk oral HPV (N = 673).

Sociodemographic characteristics, sexual history and periodontal status	Any high-risk oral HPV		Unadjusted OR
	Yes	No***	Yes
	n = 40 (%)	n = 633 (%)	n = 40 (%)
Age			
≤40	27 (7.9)*	313 (92.2)	**2.12 (1.08–4.19)**
>40	13 (3.9)	320 (96.1)	Ref
Gender			
Male	13 (6.1)	199 (93.9)	1.05 (0.53–2.08)
Female	27 (5.9)	434 (94.1)	Ref
Location			
Non-metropolitan	17 (3.8)	427 (96.2)	Ref
Metropolitan	23 (10.0)*	206 (89.9)	**2.80 (1.47–5.36)**
Completed education level¥			
High school or less	24 (5.5)	416 (94.5)	Ref
University or further	15 (6.8)	206 (93.2)	1.26 (0.65–2.46)
Tobacco smoking¥			
Yes	28 (5.7)	467 (94.3)	0.94 (0.45-.99)
No	10 (5.9)	158 (94.1)	Ref
Alcohol intake			
Ex-drinker/Current	37 (6.0)	577 (93.9)	1.19 (0.36–4.01)
Never	3 (5.1)	56 (94.9)	Ref
History of oral sex¥			
Yes	29 (7.5)	359 (92.5)	2.05 (0.92–4.57)
No	8 (3.8)	203 (96.2)	Ref
Periodontitis			
Yes	7 (6.1)	108 (93.9)	1.03 (0.44–2.39)
No	33 (5.9)	525 (94.1)	Ref

*significant p value for chi square

*** includes those with no HPV detected and those with low risk subtypes

¥ Missing values

**Table 3:** Bivariate association between sociodemographic and periodontitis with HPV 16 and/or 18 (N = 673).

Sociodemographic characteristics, sexual history and periodontal status	HPV 16 and/or 18		Unadjusted OR
	Yes	No****	n = 14 (%)
	n = 14 (%)	n = 659 (%)	
Age			
≤40	11 (3.2)**	329 (96.8)	**3.67 (1.02–13.30)**
>40	3 (0.9)	330 (99.1)	Ref
Gender			
Male	5 (2.4)	207 (97.6)	1.20 (0.40–3.63)
Female	9 (1.9)	452 (98.1)	Ref
Location			
Non-metropolitan	6 (1.3)	438 (98.7)	2.64 (0.91–7.71)
Metropolitan	8 (3.5)	221 (96.5)	Ref
Education¥			
Primary/secondary	6 (1.4)	434 (98.6)	Ref
Higher	8 (3.6)	213 (96.4)	2.71 (0.93–7.92)
Regular smoker¥			
Yes	11 (1.8)	165 (98.2)	1.25 (0.34–4.53)
No	3 (2.2)	484 (97.8)	Ref
Alcohol intake			
Ex-drinker/Current	13 (2.1)	601 (97.9)	1.25 (0.16–9.76)
Never	1 (1.7)	58 (98.3)	Ref
History of oral sex¥			
Yes	10 (2.6)	378 (97.4)	2.76 (0.60–12.74)
No	2 (0.9)	209 (99.1)	Ref
Periodontitis			
Yes	3 (2.6)	112 (97.4)	1.33 (0.37–4.85)
No	11 (1.9)	547 (98.1)	Ref

**significant p value for fisher exact

**** includes those with no HPV detected, those with low risk types and those with high risk subtypes other than 16 and 18

¥ Missing values

**Table 4:** Adjusted association between periodontitis and any high-risk oral HPV and HPV 16 and/or 18.

Exposure	Any high-risk oral HPV (Model A)	HPV 16 and/or 18 (Model B)
Periodontitis		
Yes	1.10 (0.45–2.70)*	1.27 (0.32–5.03)*
No	Ref	Ref
AUC	72.8	81.4
Hosmer-Lemshow chi^2^ (p value)	10.35 (0.24)	12.14 (0.14)

*adjusted for age, gender, location, education, smoking and alcohol intake, and history of oral sex

## Discussion

To the best of our knowledge, this is the first study to evaluate the association between high-risk oral HPV and periodontitis among Indigenous South Australians. Overall 6.0% of participants had any high-risk oral HPV (IARC classification) and HPV 16 and/or 18 subtypes were detected among 2.1%. Our estimates of any type of high-risk oral HPV are lower than estimates reported among non-Indigenous Australians [21] and other international cohorts [20] but comparable to the prevalence seen in the baseline laboratory findings of the same cohort [23]. Similarly, less than one-fifth of participants had self-reported periodontitis, which is lower than the prevalence reported in studies with clinical assessments of periodontitis among Indigenous Australians [24], but comparable to the prevalence reported in the most recent national survey of oral health among Australian adults [25].

Our study did not find any significant association between self-reported periodontitis and the presence of any high-risk oral HPV or the specific HPV 16 and/or 18 subtypes. This is in contrast to a previous study among Hispanic individuals which found a significant correlation between oral HPV and periodontitis; however, that study focused on any type of HPV and did not report high-risk subtypes alone [20]. A further prospective study conducted in Austria among 187 adults reported a significant association between oral HPV and poor oral health. Poor oral health was defined as patients with approximal plaque index (proportion of plaque-covered interproximal spaces), gingival bleeding index (proportion of bleeding sites) of more than 40% and lifetime number of extracted teeth [26]. In both these studies, a clinical assessment was done to detect periodontitis, plaque, and gingivitis, which could have resulted in a more robust ascertainment of periodontitis than our study [20, 26]. On the other hand, a US study utilising NHANES data did not find any association between clinically detected periodontitis and oral HPV infection [27]. Studies from India and Brazil have also failed to report any significant association between HPV and periodontitis [28, 29]. A recent systematic review examining the association of periodontitis and oral HPV reported no association of periodontitis with high-risk oral HPV but a positive association with any type of oral HPV, although the quality of evidence was low [12].

The conflicting results of the published literature are likely due to methodological differences in the detection and definition of periodontitis, as some studies have used radiographic assessment, whereas others are based on a clinical assessment or self-reporting [12]. Furthermore, differences in the ascertainment of HPV status may also explain the conflicting findings, with studies using gingival samples [26, 28, 29] and an oral rinse [20] for detecting HPV. The sensitivity and specificity of HPV detection techniques are known to vary, with no current Food and Drug Administration-approved tests to detect HPV DNA or mRNA in saliva [30]. In the current study, we used a saliva sample as it is non-invasive, easy to administer, and can detect the presence of virus in all the surrounding epithelial sites [30]. In addition, variation in sample sizes and differences in baseline characteristics of participants may further explain differences in the findings of existing studies [12].

There are a number of possible mechanisms which could relate periodontitis with oral HPV persistence. Periodontitis is the result of an inflammatory response of the pathogens that leads to pocketing, alveolar bone loss, and subsequent tooth loss [31, 32]. It has been suggested that periodontal pockets provide the perfect environment for HPV to persist in the oral cavity [11, 33]. Continuous release of cytokines exacerbates the inflammation process which in turn can lead to tissue damage and could therefore modulate the proliferation of HPV [22, 34]. Thus, it is important to recognize that it is possible that direct effects of oral microbes as well as the stimulation of a chronic inflammation process could underpin an association between periodontitis and HPV.

The clear biological plausibility underpins the need for future targeted studies using validated clinical data on periodontitis to clarify the relationship between periodontitis and high-risk oral HPV. This information is essential as it would allow screening and ongoing monitoring of individuals with periodontitis to reduce the persistence of oral HPV which in turn may reduce the risk of HPV-positive OSCCs, particularly in high-risk groups such as Indigenous Australians. Moreover, the detection of high-risk oral HPV in Indigenous adults in our study reinforces the continued need for dental counselling on the importance of good oral hygiene and also the importance of HPV vaccination among Indigenous peoples, given the known efficacy of vaccination against oral HPV infection [35].

It is also recommended that dentists should advise patients about the risk of HPV and promote public awareness of oral health and HPV-related risks of cancer. As the current diagnosis of OSCCs relies on patient presentation and oral physical examination plus biopsy, most OSCCs are diagnosed at a late stage, reflected in less than optimal survival rates. As a result, new approaches to screening are required to permit early detection and management. Implementation of screening for OPSCCs as part of routine dental care should be considered. This is particularly pertinent for Indigenous peoples, who experience both a higher incidence rate and lower survival rate from OSCCs than non-Indigenous Australians.

There are several limitations that should be considered when interpreting the findings of this study. First, participants were not representative of the entire Australian Indigenous population, therefore, findings may not be generalizable. Second, the cross-sectional nature of data collection limits examination of temporality, and possible causal relationships. Third, a self-reported questionnaire was used to collect information from sociodemographic variables and also for assessing periodontitis and could be subject to recall bias. Fourth, clinical assessment of periodontitis was not possible due to restrictions on travel and clinical consultations during the COVID-19 pandemic. Therefore, a non-validated self-reported tool was the best available assessment. Strict criteria were used to define periodontitis, through the inclusion of questions that have been shown to have high sensitivity and specificity for detecting periodontitis among non-Indigenous Australians. As a result, it is possible that our criteria only identified participants with moderate to severe periodontitis, which may explain our null findings. Nevertheless, clinical and radiographical assessments of periodontitis are costly, time-consuming, and often not feasible in large population-based studies. Further exploration and validation of self-reported measures of periodontal disease is an important area of future research to permit lower-cost population assessment of periodontitis. Finally, the types of HPV found in the mouth are almost entirely sexually transmitted. Several sexual behaviours could affect the transmission of HPV in the mouth for example number of sexual partners, unprotected sex, and age at first sex [36]. However, in the current study, we only adjusted for history of any oral sex as this is one of the main routes of acquiring an oral HPV infection. While this study has incorporated all the important confounding variables as well as sensitive risk behavioural data, the possibility of residual confounding cannot be excluded.

## Conclusion

This study did not find any association between self-reported periodontitis and high-risk oral HPV among Indigenous South Australians. Further studies using the clinical diagnosis of periodontitis are needed to evaluate the role of periodontitis in the acquisition and persistence of high-risk oral HPV, as this may help in identifying individuals at high-risk of oncogenic HPV and thus OPSCC.

## Supporting information

S1 Checklist STROBE statement—checklist of items that should be included in reports of *cross-sectional studies*. (DOC)

S1 Fig Goodness of fit. (DOCX)
