# Supplementary material for: Association between self-reported periodontitis and high-risk oral human papillomavirus infection among Indigenous South Australians: A cross-sectional study
Source: PLoS One. 2022 Mar 24;17(3):e0265840. doi: 10.1371/journal.pone.0265840 (PMC8946737; doi:10.1371/journal.pone.0265840)
Supplement: S1 Fig — (DOCX) [file pone.0265840.s002.docx]

# Supporting Information

**S1 Fig: Goodness of fit**

Model A Model B
